# Developing machine learning models to predict multi-class functional outcomes and death three months after stroke in Sweden

**DOI:** 10.1371/journal.pone.0303287

**Published:** 2024-05-13

**Authors:** Josline Adhiambo Otieno, Jenny Häggström, David Darehed, Marie Eriksson

**Affiliations:** 1 Department of Statistics, USBE, Umeå University, Umeå, Sweden; 2 Department of Public Health and Clinical Medicine, Sunderby Research Unit, Umeå University, Umeå, Sweden; University of Kragujevac, SERBIA

## Abstract

Globally, stroke is the third-leading cause of mortality and disability combined, and one of the costliest diseases in society. More accurate predictions of stroke outcomes can guide healthcare organizations in allocating appropriate resources to improve care and reduce both the economic and social burden of the disease. We aim to develop and evaluate the performance and explainability of three supervised machine learning models and the traditional multinomial logistic regression (mLR) in predicting functional dependence and death three months after stroke, using routinely-collected data. This prognostic study included adult patients, registered in the Swedish Stroke Registry (Riksstroke) from 2015 to 2020. Riksstroke contains information on stroke care and outcomes among patients treated in hospitals in Sweden. Prognostic factors (features) included demographic characteristics, pre-stroke functional status, cardiovascular risk factors, medications, acute care, stroke type, and severity. The outcome was measured using the modified Rankin Scale at three months after stroke (a scale of 0–2 indicates independent, 3–5 dependent, and 6 dead). Outcome prediction models included support vector machines, artificial neural networks (ANN), eXtreme Gradient Boosting (XGBoost), and mLR. The models were trained and evaluated on 75% and 25% of the dataset, respectively. Model predictions were explained using SHAP values. The study included 102,135 patients (85.8% ischemic stroke, 53.3% male, mean age 75.8 years, and median NIHSS of 3). All models demonstrated similar overall accuracy (69%–70%). The ANN and XGBoost models performed significantly better than the mLR in classifying dependence with F1-scores of 0.603 (95% CI; 0.594–0.611) and 0.577 (95% CI; 0.568–0.586), versus 0.544 (95% CI; 0.545–0.563) for the mLR model. The factors that contributed most to the predictions were expectedly similar in the models, based on clinical knowledge. Our ANN and XGBoost models showed a modest improvement in prediction performance and explainability compared to mLR using routinely-collected data. Their improved ability to predict functional dependence may be of particular importance for the planning and organization of acute stroke care and rehabilitation.

## Introduction

Stroke is the third-leading cause of death and disability worldwide [1]. Functional disabilities, cognitive deficits, and emotional problems are common after stroke [1, 2]. In addition to severe consequences to the patient, stroke is one of the costliest diseases, and more accurate predictions of stroke outcomes can guide healthcare organizations in allocating appropriate resources to reduce both the economic and social burden of the disease. Also, clinicians and other healthcare providers are guided on different policies that involve treatment and rehabilitation care for stroke patients.

Recently, models based on modern machine learning (ML) algorithms have shown potential in predicting stroke [3–6], and cardiovascular diseases [7]. Compared to traditional regression models, these algorithms can learn from data by exploring flexible relationships between a high number of predictive features and the outcome variable to achieve high prediction accuracy [8, 9]. Previous studies on stroke outcome prediction have focused on ‘unfavorable’ or ‘favorable’ functional outcomes, using 30-day mortality [5, 6] or different cut-offs [4–6, 10, 11]. For instance, some studies have used a binary version of modified Rankin Scale (mRS) with two classes: good (mRS ≤2) and poor (mRS >2) outcomes [12–14]. Ordinal analysis of the three-month mRS is more useful, since it better captures the spectrum of outcomes after stroke [10, 15].

In health care applications, a lack of studies using ML to predict death and disability as a multi-class outcome after stroke, and the black-box nature of ML models leading to difficulties in interpreting the decision process are challenges [16, 17]. However, modern ML models may provide explainability that is comparable to traditional models and domain knowledge interpretation [18]. One study suggested that explainability can help patients to make informed decisions together with clinicians, and contributes to fair distribution of available resources, while a lack of “explainability in clinical decision support systems poses a threat to core ethical values in medicine” [19].

The main aim of this study was to develop and evaluate the performance of ML models—compared to traditional multinomial logistic regression (mLR) in predicting death and functional outcomes three months after stroke—using mRS as a multi-class outcome and based on information from a nationwide stroke registry. Another aim was to assess the explainability of these models.

## Methods

### Design and setting

This study was based on data from the Swedish Stroke Registry (Riksstroke), which was established in 1994 (www.riksstroke.org). It consists of all 72 hospitals in Sweden providing specialist care for acute stroke patients and includes over 90% of all stroke patients treated in hospitals [20]. The registry includes key patients’ characteristics and information from the entire chain of stroke care, such as primary prevention, acute care, rehabilitation measures, secondary prevention, and family and community support. Patient-reported outcomes, including functional status, are collected via a questionnaire three months after stroke.

**Fig 1:**
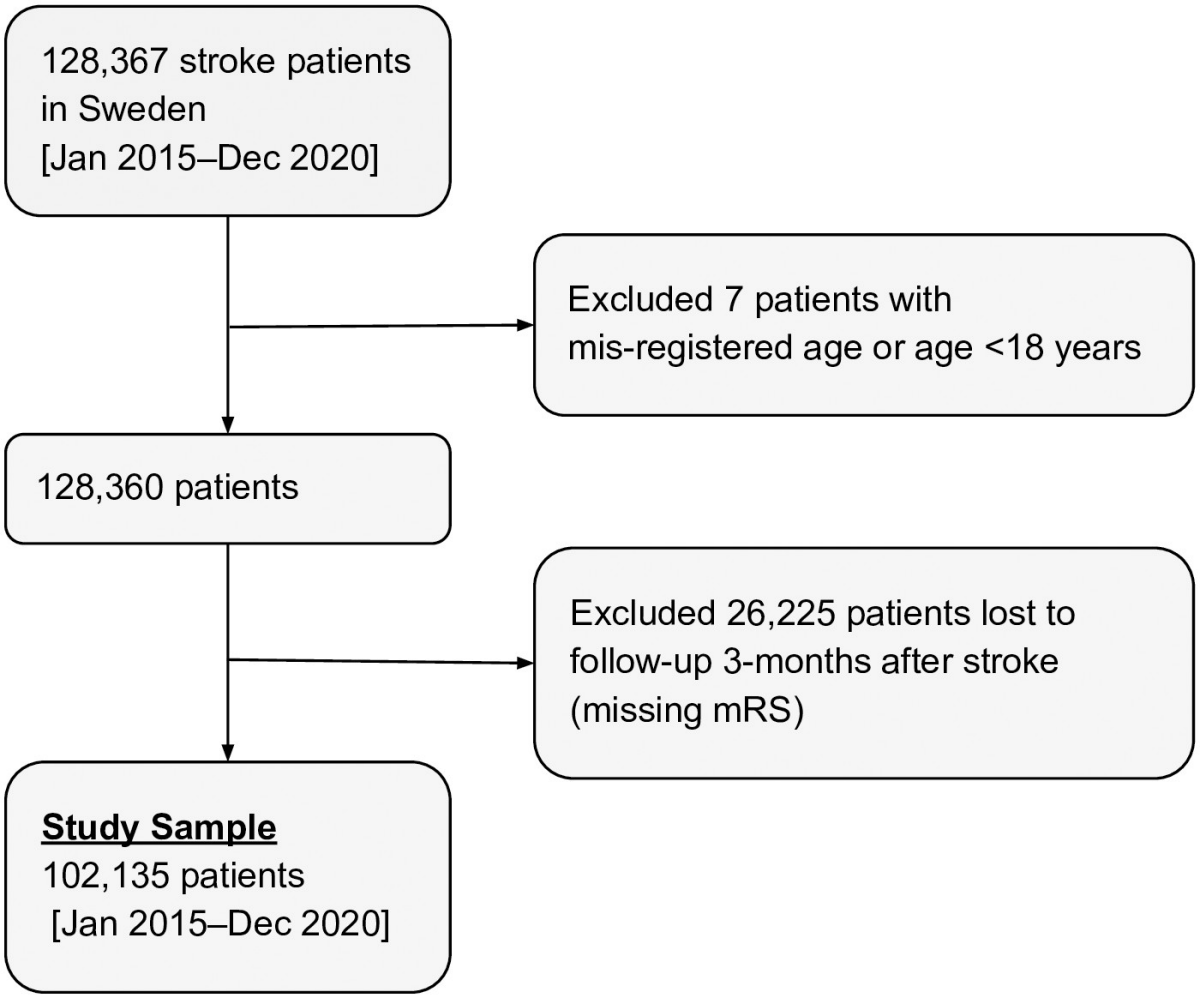
Flow chart of study sample inclusion/ exclusion criteria.

This study was observational with no risk for the study participants. To optimize model performances, adult patients who had a stroke, that is, intracerebral hemorrhage based on the International Classification of Diseases, Tenth Revision (ICD10: I61), ischemic stroke (I63), or an unspecified stroke (I64) between January 2015 and December 2020 were included. This provided a very large sample allowing outcome proportions to be estimated with high precision (low margin of error), global shrinkage factor close to 1, and small absolute difference in the apparent and adjusted Nagelkerke *R*^2^ [21, 22]. Data was accessed for the study on February 17, 2022. Excluded were all patients <18 years of age and those lost to follow-up three months after stroke, leaving a total of 102,135 patients for inclusion in the analyses (Fig 1).

This study was approved by the Swedish Ethical Review Authority (reference number 2021-06152-01). Data was pseudonymized by Riksstroke before it was submitted for the study. Patients are informed about registration in the quality registry Riksstroke that the registry aims to support high and consistent quality of care for stroke patients throughout Sweden, and that data may be used for research purposes. In accordance with the Personal Data Act (Swedish law No. SFS 1998:204), no informed consent is needed to collect data from medical charts or inpatient records for quality registries. However, patients are informed of their rights to deny participation (opt-out consent). The study is reported in agreement with both the Transparent Reporting of a multivariable prediction model for Individual Prognosis Or Diagnosis (TRIPOD) checklist for prediction model development and validation, and the Strengthening the Reporting of Observational Studies in Epidemiology (STROBE) checklist for cohort studies [23, 24].

### Variables

The primary outcome for prediction was mRS at three months of stroke, which was categorized into three levels (0–2 independent, 3–5 dependent, and 6 dead). Since mRS is not measured directly in Riksstroke, a previously-developed algorithm was used where self-reported Riksstroke outcome questions on dependency, living situation, mobility, dressing, and toileting have been shown to be translatable into mRS grades with high precision [25]. The outcome of interest could not be assessed blindly considering that the study was based on information already collected in Riksstroke. Besides, the expected proportions of death and dependency are well known.

Different prognostic factors (features) for prediction included age (years), sex, cardiovascular risk factors (atrial fibrillation (AF), diabetes, previous stroke or transient ischaemic attack (TIA), and smoking status), medications before stroke (treatment for high blood pressure, lipid-lowering drugs, and anticoagulants), modified Rankin Scale (mRS) prior to the stroke (levels: 0–2, 3, 4, 5) [25], stroke severity at admission (measured by the National Institutes of Health Stroke Scale (NIHSS) (levels: 0–42)), type of stroke (ischemic, hemorrhage, unspecified), wake-up stroke, arriving at the hospital by ambulance, stroke alert activation (prehospital notifications from ambulances and alerts at the emergency department for patients presenting with stroke symptoms), inpatient at the time of stroke, the hour of admission (band by 4), and day of the week [26, 27] the patient was admitted to the hospital. These features were selected based on previous knowledge and availability in the registry.

### Data analysis

Data preprocessing was conducted using R 4.1.1 [28], and all other analyses were performed in Python 3.7.4 [29]. The same features were included in all models.

#### Label encoding

Age and NIHSS were analyzed as continuous variables. To improve predictions and performance, nominal variables were transformed using one-hot encoding where each level of the nominal variable was converted into a new binary variable [30]. Binary variables were coded as 1 (Yes) if they matched the level and 0 (No) otherwise (reference category).

#### Missing data

NIHSS had a large proportion of missing data, and multivariate imputation by chained equations (MICE) algorithm was implemented [31, 32]. A single imputed dataset (*m* = 1) was considered due to the challenge of pooling results from a ML model. For instance, the process of training a ML model on several imputed datasets could be computationally too expensive. Level of consciousness at hospital admission (alert, drowsy, unconscious), together with the variables included in this study, were used to impute NIHSS. Level of consciousness was then excluded from further analysis considering that it was part of the NIHSS scores as a measure of stroke severity [33, 34]. The imputation was evaluated by density plots comparing the distribution of NIHSS in imputed and observed data. For all other variables, which had low proportions of missingness, a separate category was assigned to the missing values.

#### Feature scaling

Features with larger numeric ranges (e.g., continuous variables) compared to others, may either dominate the prediction algorithm or cause computational problems. To equalize the feature contribution to the model training, an interval scaling method known as Min–Max scaling was used to transform the feature values to a range of [0, 1] [35, 36].

#### Classification models

The ML models (SVM, ANN, and eXtreme Gradient Boosting (XGBoost)) were developed and compared to both main effects mLR and mLR with the addition of all possible 2-way interaction terms in terms of their predictive performances on the mRS. These models were chosen due to their widespread application in medicine and previously demonstrated performances in classification [11, 37, 38]. A brief outline of these algorithms is provided in S1 Method.

#### Model training process

Models were trained and evaluated on the data which was randomly split into 75% training and 25% test sets, maintaining the same outcome proportions in both sets. The very large sample and random split, ensures minimal differences between training and test set (data not shown). The reference model was fitted using the ‘*LogisticRegression*’ classifier from the Sklearn library [39]. For the ML models, an initial set of values for some hyperparameters (e.g., maximum depth of trees, kernel, size of hidden layers among others) were explicitly specified while others were set to default values because their default values were either appropriate (e.g., booster, activation function, initial learning rate) or were less susceptible to hyperparameter space (e.g., size of the kernel cache, maximum number of iterations) [40]. We then implemented a grid search algorithm using 5-fold cross-validations (CVs) based on *f1_weighted* score. The algorithm performs an exhaustive search on these hyperparameters to estimate an optimal combination of the parameters, thus improving the model’s prediction accuracy as well as preventing model overfit [41]. In the end, the optimal model was evaluated on the reserved 25% test set to obtain its prediction performance.

#### Model performance metrics

After model development, we evaluated their predictive performances using different performance metrics for multi-class outcomes such as recall, precision, accuracy, Cohen’s Kappa, Matthew’s correlation coefficient (MCC), F1 score, and area under the receiver operating characteristic curve (AUC–ROC) [42]. All these measures are based on the confusion matrix (S2 Method and S2 Fig).

The estimates of these metrics were reported together with their 95% confidence intervals (CIs). A non-parametric bootstrap resampling method was applied to compute the CIs, where a total of 1000 patient-wise resampling of size 25,534 (test set sample size) was carried out on both the true outcome and predicted values obtained from the test dataset with replacement. The performance metric for each bootstrap sample was calculated and the percentile with 95% confidence intervals for the estimates obtained. All the metrics have been implemented in Sklearn [39].

#### Model explainability

In general, modern ML models do not provide measures to quantify or describe the contribution of each covariate on the outcome, and this could be a drawback in terms of clinical utility compared to e.g., LR [43]. To increase explainability and interpretability, we obtained SHAPley Additive exPlanations (SHAP) values of each feature, as implemented in the SHAP Python library [44]. The values are calculated based on the formula in S3 Method. In this study, the results are presented using two graphical methods, including a force plot that explains the feature importance at a patient level (local explainability), and a beeswarm plot that illustrates a visual representation of feature-specific SHAP values across all patients (global explainability) (S3 Method).

## Results

### Summary statistics

Of the 102,135 eligible patients, 87,594 (85.8%) had an ischemic stroke. The mean [standard deviation (SD)] age at stroke onset was 75.8 [12.0] years, a median NIHSS score [Q1-Q3] of 3 [1–8], and 54,473 (53.3%) were males (Table 1). Missing data accounted for 1.3% of the whole dataset, with the highest level of missingness in the NIHSS variable (42.6%), while missing data for other features ranged between 0% to 18.3%. The imputed NIHSS values had a distribution similar to the observed data (S1 Fig), which indicate that the MICE imputation worked well. Patients lost to follow-up were on average 3 years younger, were more often males (55.3% vs. 53.3%), and had a similar stroke severity, despite a slightly worse risk factor profile (S1 Table).

**Table 1:** Summary of patient characteristics (demography, cardiovascular risk factors, primary preventive medication, and stroke characteristics) categorized by mRS. Reported descriptive statistics including mean, standard deviation (SD), median, quartiles (Q1, Q3), and frequencies (number of patients) (%). The last column shows the proportion of missing cases in each of the variables (features).

	Outcomes 3 months after stroke				Missing (%)
	All patients	mRS 0–2	mRS 3–5	mRS 6	
**Number of patients (%)**	102,135 (100)	43,361 (42.5)	36,687 (35.9)	22,087 (21.6)	
Age (mean [SD])	75.8 [12.0]	70.4 [11.6]	78.7 [10.8]	81.8 [10.3]	-
Male (%)	54,473 (53.3)	26,797 (61.8)	17,328 (47.2)	10,348 (46.9)	-
Atrial Fibrillation (%)	30,195 (29.6)	8,315 (19.2)	12,234 (33.3)	9,646 (43.7)	152 (0.2)
Diabetes (%)	22,355 (21.9)	7,759 (17.9)	9,358 (25.5)	5,238 (23.7)	214 (0.2)
Previous Stroke or TIA (%)	27,787 (27.2)	8,505 (19.6)	12,013 (32.7)	7,269 (32.9)	186 (0.2)
Smoking (%)	11,394 (11.2)	5,852 (13.5)	4,020 (11.0)	1,522 (6.9)	14,252 (14.0)
Blood pressure (BP) lowering medication (%)	65,004 (63.6)	24,938 (57.5)	25,076 (68.4)	14,990 (67.9)	395 (0.4)
Lipid-lowering drugs (%)	32,616 (31.9)	13,473 (31.1)	12,638 (34.4)	6,505 (29.5)	441 (0.4)
Prior anticoagulation (%)	15,824 (15.5)	4,526 (10.4)	6,423 (17.5)	4,875 (22.1)	318 (0.3)
Pre-stroke mRS (%)					5,199 (5.1)
0–2 (Reference)	69,795 (68.3)	41,484 (95.7)	19,977 (54.5)	8,334 (37.7)	
3	14,772 (14.4)	1,416 (3.3)	9,233 (25.2)	4,123 (18.7)	
4	9,352 (9.2)	200 (0.5)	4,857 (13.2)	4,295 (19.4)	
5	3,017 (3.0)	2 (0.0)	1,102 (3.0)	1,913 (8.7)	
Inpatient at time of stroke (%)	6,184 (6.1)	1,426 (3.3)	2,211 (6.0)	2,547 (11.5)	2 (0.0)
NIHSS at arrival (median [Q1-Q3])	3.0 [1.0–8.0]	2.0 [0.0–4.0]	4.0 [2.0–9.0]	13.0 [6.0–20.0]	43,474 (42.6%)
Type of stroke (%)					
Ischemic (Reference)	87,594 (85.8)	39,720 (91.6)	31,905 (87.0)	15,969 (72.3)	-
Hemorrhagic	13,511 (13.2)	3,282 (7.6)	4,492 (12.2)	5,737 (26.0)	-
Unspecified	1,030 (1.0)	359 (0.8)	290 (0.8)	381 (1.7)	-
Wake-up stroke (%)	18,635 (18.2)	8,683 (20.0)	6,495 (17.7)	3,457 (15.7)	18,729 (18.3)
Stroke alert activation (%)	35,277 (34.5)	14,014 (32.3)	12,883 (35.1)	8,380 (37.9)	1,319 (1.3)
Ambulance service to the hospital (%)	70,786 (69.3)	24,349 (56.2)	28,180 (76.8)	18,257 (82.7)	8,469 (8.3)
Hour of Admission (%)					4,021 (3.9)
00–04 (Reference)	4,332 (4.2)	1,723 (4.0)	1,508 (4.1)	1,101 (5.0)	
04–08	5,852 (5.7)	2,553 (5.9)	1,891 (5.2)	1,408 (6.4)	
08–12	29,094 (28.5)	12,591 (29.0)	10,127 (27.6)	6,376 (28.9)	
12–16	27,870 (27.3)	12,371 (28.5)	10,317 (28.1)	5,182 (23.5)	
16–20	19,396 (19.0)	8,204 (18.9)	7,190 (19.6)	4,002 (18.1)	
20–24	11,570 (11.3)	4,632 (10.7)	4,219 (11.5)	2,719 (12.3)	
Day of week of admission (%)					44 (0.04)
Sunday (Reference)	13,214 (12.9)	5,290 (12.2)	4,951 (13.5)	2,973 (13.5)	
Monday	16,255 (15.9)	7,126 (16.4)	5,806 (15.8)	3,323 (15.0)	
Tuesday	15,377 (15.1)	6,712 (15.5)	5,435 (14.8)	3,230 (14.6)	
Wednesday	15,147 (14.8)	6,511 (15.0)	5,446 (14.8)	3,190 (14.4)	
Thursday	14,740 (14.4)	6,348 (14.6)	5,263 (14.3)	3,129 (14.2)	
Friday	14,446 (14.1)	6,149 (14.2)	5,105 (13.9)	3,192 (14.5)	
Saturday	12,912 (12.6)	5,210 (12.0)	4,652 (12.7)	3,050 (13.8)	

### Tuning model hyperparameters

S2 Table displays the selected hyperparameters that were tuned with their grid search ranges to achieve the best parameter combination.

### Model prediction performance

**Fig 2:**
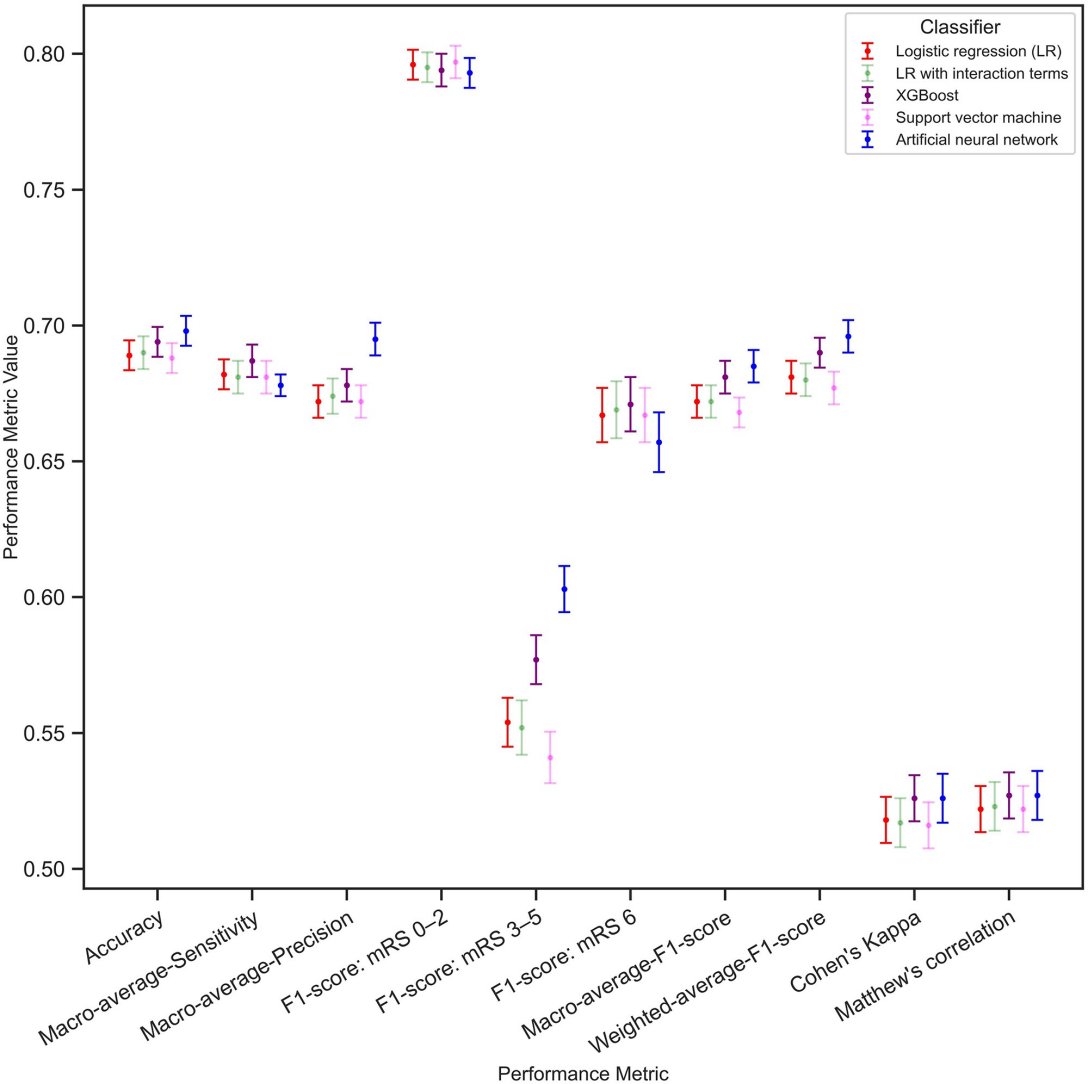
Performance metric values with their corresponding 95% confidence intervals for each classifier multinomial logistic regression (mLR), mLR with 2-way interaction terms, XGBoost, SVM, and ANN). The *x*-axis shows different performance metrics with their corresponding values on the *y*-axis. Each dot is the estimated value of the metric while the lines denote the 95% confidence intervals within which the value lies.

Overall, all models were able to correctly predict the mRS levels in more than 68% of the patients (Fig 2 and S3A Table). There were no significant differences in the performance metric values between mLR and mLR with 2-way interaction terms. Accuracy gives relatively higher weights to the predictive performance of the most frequent outcome category (i.e., independence). On the other hand, the F1 scores give similar or higher weights to smaller-sized categories (independence and death). The performance of the ANN model compared to mLR showed a significant improvement with a weighted average F1 score of 0.696 (95% CI; 0.690–0.702) versus 0.681 (95% CI; 0.675–0.687), and a macro average precision of 0.695 (95% CI; 0.689–0.701). Similarly, both ANN and XGBoost models demonstrated significantly better performances compared to the mLR in correctly classifying the functional dependence group with respective F1 scores of 0.603 (95% CI; 0.594–0.611) and 0.577 (95% CI; 0.568–0.586), versus 0.554 (95% CI; 0.545–0.563).

The accuracy values ranged between 0.68–0.70 with overlapping confidence intervals in both the training and test sets. This shows a low degree of overfitting in the training data (S3A and S3B Table).

S3 Fig displays AUC-ROC curves based on a one-versus-rest strategy for mRS categories. On average, all the ML models performed better than the mLR in classifying patients into the mRS categories, and an improvement of more than 10% is observed in both ANN and XGBoost versus mLR in classifying the functional dependent group.

### Model explainability

Since Odds Ratios (ORs) of LR are interpretable, we present their estimates in S4 Table. The ORs from mLR express the ratio of the odds of the covariate among patients in the corresponding mRS level to the odds of the covariate among patients in the other two mRS levels, holding all other covariates constant. For instance, a one-year increase in age is associated with a decrease in the odds of belonging to the functional independent group by 4.2%, and the same change in age increases the odds of dying by 4.0% (S4 Table).

**Fig 3:**
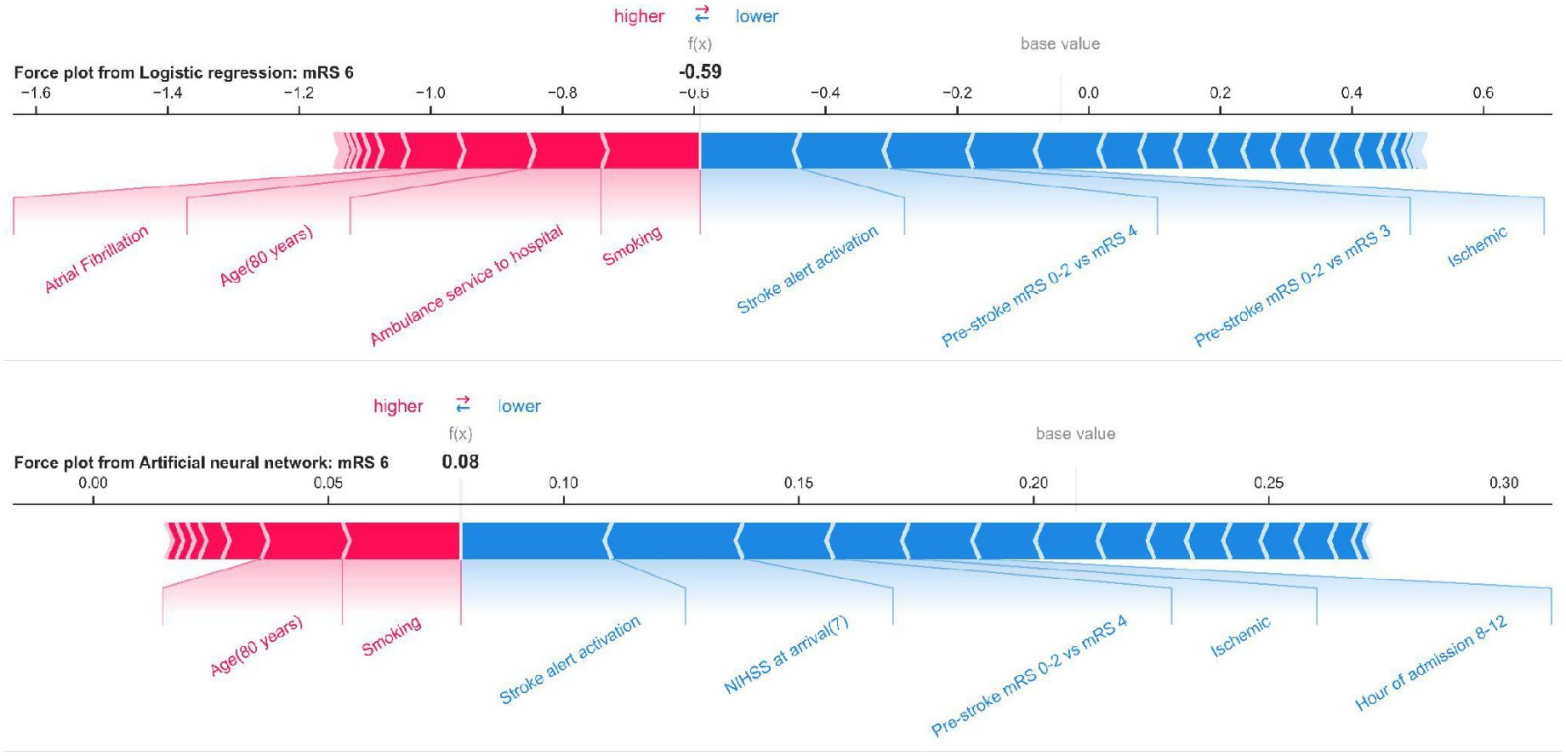
SHAP force plot of predictions from mLR and ANN classifiers for death at a patient level. The colors show whether the feature increases (red) or decreases (blue) the probability of death for this patient. Bar size denotes the SHAP value. The longer the bar the greater the influence on prediction. The base value indicates the average prediction (with no feature in the model) of the model (log odds) based on the training data, while *f(x)* is the final predicted probability of the mentioned patient to belong to this outcome class (death).

Fig 3 illustrates local explainability by a SHAP force plot for a typical patient with the following features: female sex, aged 80 years, ischemic stroke, NIHSS score of 7, functionally independent before stroke, received blood pressure lowering drugs, had atrial fibrillation, a smoker, had stroke alert activation, arrived at hospital by ambulance, and was admitted on a Wednesday between 8–11.59 a.m. The colors show whether the feature increases (red) or decreases (blue) the probability of predicting that outcome class. Bar size denotes the SHAP value. The longer the bar the greater the influence on prediction. The base value indicates the average prediction of the model (log odds) based on the training data, while *f*(*x*) is the final predicted probability (given in log odds) of the patient belonging to that outcome class. It is observed that for this specific patient’s prediction, smoking, arriving at the hospital by ambulance, age, and atrial fibrillation, among others increased the risk of death, while stroke alert activation and functional independence before the stroke among others reduced the risk of death.

**Fig 4:**
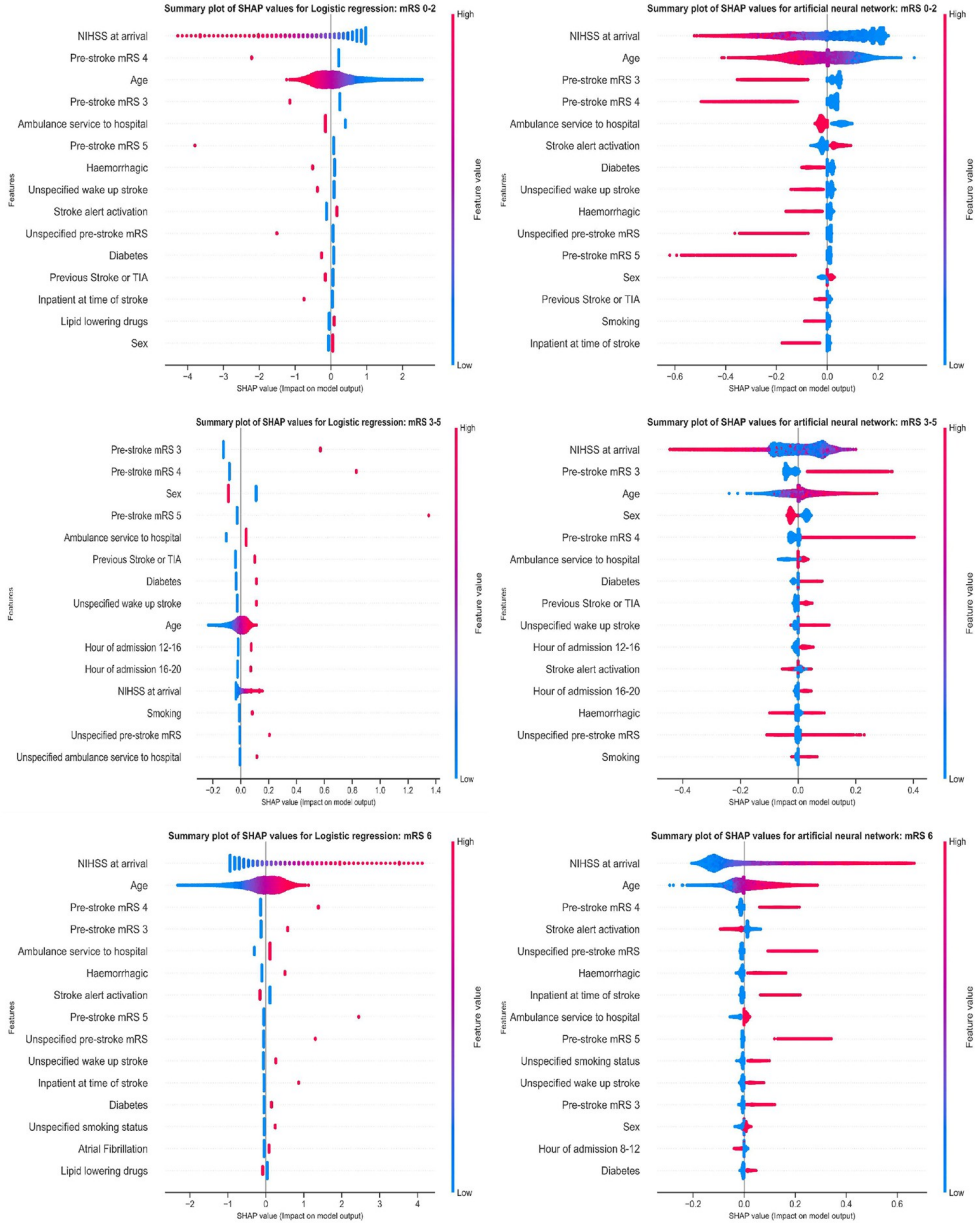
SHAP plots for mLR and ANN classification models for each mRS category. The features are given in order of importance together with the corresponding mean absolute SHAP value. For each feature, a patient is represented by a point (dot) distributed horizontally along the *x*-axis based on the SHAP value. Red and blue points are the high (male sex or yes for other binary features) and low (female sex or no for other binary features) feature values, respectively. The horizontal axis indicates the feature effect on prediction (either increasing or decreasing the probability of belonging to that outcome class).

Fig 4 displays global explainability by SHAP plots for each mRS class obtained from the reference LR model and the ANN model (plots for other models are shown in S4 Fig). The features are given in order of importance (descending mean absolute SHAP values). Points are distributed horizontally along the *x*-axis based on the patient’s SHAP values, with overlapping points jittered in the *y*-axis direction. The red and blue points are the high (male sex or yes for other binary features) and low (female sex or no for other binary features) feature values, respectively. The horizontal axis indicates the feature effect on prediction (either decreases or increases the probability of being in an outcome class). We observed that predictions of independence in functional outcome were related to male sex, lipid-lowering drugs, and activation alerts. While NIHSS, age, ambulance usage, stroke type hemorrhage, pre-stroke mRS, inpatient at time of stroke, atrial fibrillation, diabetes, and male sex increase the risk of death. Level of NIHSS contributed the most to all predictions of all mRS groups in all models except when mLR was used to model dependency.

## Discussion

In this prognostic study based on nationwide registry data, we developed three ML prediction models and compared their performance to that of the traditional mLR model. Overall, our models that were based on ANN and XGBoost demonstrated a modest improvement in predicting multi-class outcomes (independence, dependence, or death) three months after stroke compared to mLR. Also, the former models were significantly better than mLR in predicting functional dependence, which was the most difficult outcome to predict. This could be of relevance for continued care, rehabilitation planning, and estimation of cost-efficiency, among others.

Furthermore, this study demonstrated how existing methods (like SHAP values) can be used to interpret and explain predictions of stroke outcomes made by complex machine learning models. As expected from previous studies [14, 45, 46], NIHSS was the most important predictor of outcomes.

Past studies on ML and stroke outcomes have mainly focused on binary outcomes [5, 10, 12, 13, 47, 48] whereas our study has an outcome with three classes. In agreement with our findings, ML (deep neural network and XGBoost in particular) have been shown to improve the prediction of long-term (mainly binary) outcomes in ischemic stroke patients [12, 15, 47]. In a registry-based study, ANN showed an accuracy of 80.15%, which was significantly better than binary LR in predicting poor prognosis (mRS >2) after acute stroke [47]. Similarly, deep neural networks (ANN with many hidden layers) achieved a ROC-AUC value of 0.888 versus that of binary LR (AUC 0.849) in classifying outcomes (mRS ≤2 versus mRS >2) in acute ischemic stroke [12]. When restricted to features available at admission, machine learning predictions have shown limited improvements to that of scores designed by medical experts to predict functional outcomes after stroke [13]. However, for all the mentioned studies, a binary classification cannot fully capture the outcome after stroke. Also, compared to our study, the studies referenced above included relatively small sample sizes, they were single centered, and only included ischemic stroke patients. This may make it difficult to generalize these models to other settings. On a further note, a recent study demonstrated that XGBoost (AUC 0.895) performed relatively better than binary LR and a regularized binary LR with or without interaction terms in classifying all-cause 30-day in-hospital mortality after stroke in the UK [5]. Although this was based on a binary outcome and considered only one ML-based model, the findings are consistent to our study. The study included a large sample size and was based on high quality clinical registry data. An external validation of the models showed that the developed model could be generalized to the Swedish setting [6].

We extended the measure of outcome to predict a multi-class outcome including independence, dependence, and death, based on the mRS, a highly clinically relevant and common measure of functional outcome in stroke studies [49]. ANN also showed good performance in classifying the dependent functional outcome which is an important endpoint in stroke care.

We also accounted for the types of strokes in all the models. Ischemic and hemorrhagic strokes have different pathophysiology and treatment strategies. However, they also share several risk factors, treatment and rehabilitation measures, and outcome factors. With traditional modeling (e.g., mLR), there is a need to include high-order interactions to fully account for these similarities and differences. Flexible machine learning models allowed us to develop a prediction model that can use all data, taking into consideration factors that may or may not have the same effect on outcome after ischemic and hemorrhagic stroke. This would increase the power and can provide more accurate predictions.

A recent review of ML models for stroke outcome prediction reports limitations in study sample size, reporting standards, model transparency, and interpretation [11]. Our models were based on information from Riksstroke, which is estimated to include more than 90% of all stroke patients treated in hospitals in Sweden, with high validity and reliability of recorded information [20]. Due to hospital crowding, stroke patients may occasionally be treated in wards where stroke patients are not normally expected to be found. Those patients are more likely to miss registration. We built and reported our models using robust approaches including cross-validation, train–test split evaluation, use of SHAP values, and adherence to  TRIPOD guidelines [23]. In addition, the developed models are available to researchers in the GitHub repository [https://github.com/Josline90/-Machine-Learning-Prediction-Model-of-mRS-levels.git], providing transparency and facilitating external validations of the ML models in other regions and healthcare settings.

In our study, the features underlying the predictions were limited to information that is routinely collected in electronic health records at hospital admission. On a group level, the ML prediction models developed in this study can be used to predict outcome for a patient population with a given case-mix without underlying model assumptions, hence allowing fair comparisons of outcome between treatment regimens, hospitals and regions. Machine learning models, including an extension of the mLR model to include regularization and interaction terms, provide an opportunity to include more detailed information and thereby improve predictions [13]. Including more detailed information on patient frailty, imaging results, and acute care could further improve predictions. A vision for the future could be to implement systems utilizing machine learning models in the hospitals to help predict outcomes in real-time, which could serve as a guide for clinicians when deciding on acute treatments and rehabilitation measures on the individual level.

## Limitations

This study also has some limitations. We only included information available in routine care, recorded in the Riksstroke register. Hence, the data used for analyses did not include information on some features such as imaging or congestive heart failure, among others which may also be important risk factors for predicting functional outcomes of stroke. This information is not recorded in the Riksstroke registry. Patients lost to follow-up were excluded, which may cause selection bias. Since patients who were still alive three months after stroke and did not answer the three-month follow-up questionnaire were excluded and as death is robustly reported, the analysis datasets include a higher proportion of deaths than that observed in the general stroke population. A comparison of background characteristics of patients with complete outcome at 3-months follow-up versus those who were still alive but lost to follow-up, showed minor differences, which are unlikely to have a major impact on model performance or explainability.

Stroke severity, measured by NIHSS included a high proportion of missing, and MICE was used for imputation. The distribution of imputed and observed data were similar, which suggest that the imputed values were plausible [50]. This is in agreement with the previous finding that MICE performs better compared to deep learning imputation methods such as generative adversarial imputation network (GAIN), and multiple imputation using denoising autoencoders (MIDA) [51, 52]. Comparisons of model performance include several aspects. Here, we chose to impute missing NIHSS values prior to model training and testing. This allowed more straightforward comparisons, but does not incorporate an evaluation on how e.g., several ML approaches can handle missing data by themselves.

The training process of the ML algorithms was computationally intensive. We decided to tune a limited number of parameters with a smaller range of values based on 5-fold CVs. However, these models are known to be robust for misspecifications [53]. A previous study showed that a model predicting all-cause 30-day in-hospital mortality after stroke that was developed in the UK could be generalized to Swedish stroke patients [6]. However, the models in the current study were not externally validated and should be validated before applications to other countries and regions, in particular if they have different demographic and health care characteristics. We therefore provide open access to the developed models [https://github.com/Josline90/-Machine-Learning-Prediction-Model-of-mRS-levels.git], allowing reproducibility to be tested in other countries and healthcare settings.

## Conclusions

ML models, that allow flexible relationships between predictive features and the outcome, can be developed and show potential to improve predictions of multi-class outcomes after stroke using routinely-collected data. Despite the lack of explicit parameter estimates, SHAP values contribute to an explainability that is comparable to that of traditional regression models. To ensure generalizability, the developed prediction models should be evaluated further in an independent stroke dataset.

## Supporting information

S1 Method Classification models. (PDF)

S2 Method Model performance metrics. (PDF)

S3 Method Model explainability. (PDF)

S1 Table Comparison of patients’ characteristics at three months follow-up. Summary statistics reported as number of patients (%) for binary variables, median (quartiles) for categorical variables, and mean (standard deviation) for continuous variables.
(PDF)

S2 Table Model hyperparameters search space (selected based on best guess). The best hyperparameter values obtained from the algorithm are presented in the last column of the table. Meaning of these hyperparameters can be obtained from the SKlearn library ([1] reference for S2 Table).
(PDF)

S3 Table **A.** Estimates (with 95% confidence interval (CI)) for the Model Performance Metrics in the test set. **B.** Estimates (with 95% confidence interval (CI)) for the Model Performance Metrics in the training set).
(PDF)

S4 Table Estimated odds ratios (exp(regression slope coefficients)) from the main-effects multinomial LR model. The estimates indicate how a unit increase in the covariate is associated with the odds of belonging to a particular mRS level, keeping other covariates fixed. *OR* = 1 means that the covariate is not associated with odds of the corresponding mRS level, *OR* > 1 means that there is increased occurrence of the corresponding mRS level as the covariate increases (or at that level of covariate), and *OR* < 1 indicates that the corresponding mRS level is less likely to occur as the covariate increases (or at that level of covariate).
(PDF)

S1 Fig Marginal density plot of observed and imputed National Institutes of Health Stroke Scale (NIHSS) at arrival. (TIFF)

S2 Fig Confusion matrices of multinomial logistic regression (mLR), mLR with 2-way interaction terms, artificial neural network, XGBoost, and support vector machine, plotted for each class of mRS. (TIFF)

S3 Fig AUC-ROC curves of multinomial logistic regression (mLR), mLR with 2-way interaction terms, artificial neural network, XGBoost, and support vector machine, plotted for each class of mRS. Macro-average is the arithmetic mean of AUC of each target class (equal contribution of weight for each class) while Micro-average is averaging that accounts for the size of each target class. Since micro-average AUC-ROC is dominated by the highest frequency class, macro-averaging becomes an alternative when the performance on all the classes is equally important.
(TIFF)

S4 Fig SHAP plots for XGBoost and SVM classifiers. (TIFF)

S1 Checklist STROBE statement—Checklist of items that should be included in reports of observational studies. (DOCX)
