# Supplementary material for: Developing machine learning models to predict multi-class functional outcomes and death three months after stroke in Sweden
Source: PLoS One. 2024 May 13;19(5):e0303287. doi: 10.1371/journal.pone.0303287 (PMC11090298; doi:10.1371/journal.pone.0303287)
Supplement: S1 Method — (PDF) [file pone.0303287.s001.pdf]

S1 Method. Classification Models

Logistic Regression (LR): LR is the most widely used model for classification of clinical data [1]. Binary LR predicts the probability of occurrence of an event versus no event given a set of covariates (features). Multinomial LR (mLR) is a generalization of binary LR that handles problems with more than two possible outcomes. In this study, a mLR that models the probability of each level k of the outcome Y given a set of p predictors $X_1,..., X_p$ was fitted. This is given by:

$$P\left(Y = k | X_1 = x_1,..., X_p = x_p, \beta\right) = \frac{e^{\beta_{0k}+\beta_{1k}x_1+...+\beta_{pk}x_p}}{1+\sum_{l=1}^{K-1} e^{\beta_{0l}+\beta_{1l}x_1+...+\beta_{pl}x_p}}$$

Where $k = \{1,..., K\}$ ($K = 3$ levels: independent, dependent, dead), and β's are the parameters estimated by minimizing the negative log-likelihood loss function defined as:

$$l(\beta | Y = k, X_1 = x_1,..., X_p = x_p) = -log\left(\prod_{i=1}^{n} P\left(Y = k | X_1 = x_1,..., X_p = x_p, \beta\right)\right)$$

$$l = -\sum_{i=1}^{n}\sum_{k=1}^{K} p\left(Y_i = k | X_{1i} = x_{1i},..., X_{pi} = x_{pi}, \beta\right) log\, p\left(\widehat{Y}_i = k | X_{1i} = x_{1i},..., X_{pi} = x_{pi}, \beta\right)$$

Where n is the total number of patients in the dataset, Y_i is the true class label, and $\widehat{Y}_i$ is the predicted class label. A main effects mLR was fitted and used as a reference model for prediction. We also fitted a mLR with the addition of all possible two-way interaction terms.

Support Vector Machine (SVM): SVM is a supervised learning technique that has gained prominence in machine learning (ML) to handle data classification problems

[2]. Since it does not directly support multi-class outcomes, we considered an '*One-versus-Rest*' (OvR) strategy to break down the multi-class outcome $y \in \{0, 1, 2\}$ (0-independent, 1-dependent, 2-dead) into three binary outcomes. Hence the classifier uses three SVMs $\{0\ vs\ [1, 2],\ 1\ vs\ [0, 2],\ 2\ vs\ [0, 1]\}$. For $i = 1, ..., n$, let patient-label pairs be (x_i, y_i), $y_i \in \{1, -1\}$ (a binary label that corresponding to each x_i depending on the side of the hyperplane it belongs to), $x_i \in R^p$, n and p denote the total number of patients and features in the training set, respectively. The equation of the hyperplane can be defined as [2]:

$$y_i = w'\phi(x_i) + b$$

where $y_i = 0$ for maximum margin hyperplane, $y_i > 0$ and $y_i < 0$ for hyperplanes lying on support vectors, b is the bias (intercept), while ϕ is a function that maps vector x_i into a higher dimensional space using a kernel function. In this study, two kernel functions (sigmoid and radial basis function) were specified in the search space when tuning the hyperparameters.

We aimed to classify data by finding the maximum-margin hyperplane that separates the group of points x_i of one class from those of the other classes. It maximizes the distance between the hyperplane and the nearest point x_i (support vectors) from either group. This is achieved by solving the optimization problem expressed as:

$$min_{w,b,\zeta}\left(\frac{1}{2}w'w + C\sum_{i=1}^{n}\zeta_i\right)$$

constraint to $y_i\left(w'\phi(x_i) + b\right) \geq 1 - \zeta_i$,

for $\zeta_i \geq 0$ denoting the distance of an incorrectly classified point from its correct hyperplane, $w \in R^p$ is the vector of weights assigned to a feature, $C > 0$ is a penalty parameter.

***Artificial Neural Network (ANN)*:** ANN is a subset of ML algorithms. We used a multi-layer perceptron ANN consisting of a weighted directed graph (architecture) of interconnected nodes known as neurons (perceptrons) that is inspired by the structure of neurons in a human brain [3]. During the training process, an input layer takes in the dataset of features as an input then passes it to the hidden layer(s). Hidden layers facilitate the learning process of the network then forward the processed data to the output layer for prediction. Each neuron of a layer connects with another from the next layer through channels with a particular weight assigned to it. The sum of the neuron values multiplied by their corresponding weights becomes the input in the subsequent hidden layer. A bias value (intercept) is added to this input sum before passing it to an activation function to get output of neurons. The activated neurons transmit data to the next layer of the network. The neuron with the largest value determines the output value in the output layer. The process is repeated until the final output layer is reached. The relationship between input and output in each node can be defined as [3,4]:

$$y_{node} = f\left(\sum_{i=1}^{n} w_i x_i + b\right),$$

where y_{node} is the output value (between 0 and 1), x_i are the input values for feature i with their corresponding weights w_i, b is the bias, and $f()$ is an activation function. The final output is then compared to the true values adjusting for weights and bias to

get more accurate predictions. This optimization process was based on gradient descent optimization algorithm minimizing the loss function [3,4]:

$$l = -\sum_{i=1}^{n}\sum_{k=1}^{K}\left[y_{ik} log\left(\hat{y}_{ik}\right)\right],$$

for $K = 3$ (number of output classes: independent, dependent, dead), n is the total number of patients in the dataset, y_{ik} is the true class label, and $\hat{y}_{ik}$ is the predicted class label in the output layer. To improve the learning process, we used the default activation function, the rectified linear unit activation function (‘*ReLu*’) since it can solve the vanishing gradient problem (as the number of layers increases, partial derivative of the loss function gets closer to zero) [5]. The weights of the network do not change as the derivative gets closer to zero.

Extreme Gradient Boosting (XGBoost): XGBoost is an efficient implementation of the gradient boosting algorithm (models are trained sequentially by minimizing the errors of the previous ones) in predictive modeling by introducing regularization to prevent overfitting [6,7]. It has become a widely used ML algorithm due to its scalability and better performance in learning the non-linear relationships between the features and the outcome. Let (x_i, y_i) be the set of input features and observed outcome value for patient i, respectively. In this study, the true outcome $y_i \in \{0, 1, 2\}$ that is, 0 means patient i is independent, 1 is dependent on others, and 2 is dead within 3 months after a stroke. The connection between x_i and the predicted outcome can be expressed as:

$$\hat{y}_i = \sum_{d=1}^{D} f_d(x_i),$$

where $\widehat{y}_i$ is the predicted value of the outcome on patient i given x_i, D is the total number of decision trees, f_d is the predicted value of decision tree d. This ensemble algorithm uses gradient descent technique to create a new decision tree by minimizing the residual errors of the preceding one, then combines all to make a final prediction. In other words, a regularized objective function which contains two components (the training loss function that measures how well the model fits the training set, and the regularization term that measures the complexity of the tree to avoid overfitting) is minimized. This is in the form [6]:

$$Obj = \sum_{i=1}^{n} l\left(y_i, \widehat{y}_i\right) + \sum_{d=1}^{D} \Omega\left(f_d\right) = \left\{\sum_{i=1}^{n} l\left(y_i, \widehat{y}_i\right)\right\} + \left\{\gamma \sum_{d=1}^{D} T_d + \frac{1}{2}\lambda \sum_{d=1}^{D} \sum_{j=1}^{T} w_{dj}^{\ 2}\right\}$$

where, n is the number of cases (patients) in the training set, T_d is the number of leaves in the dth tree, γ is the regularization parameter on the number of leaves, w_{dj} is the weight (score) on the jth leaf on d^{th} tree, and λ is the L2 regularization on the leaf weights. Since our prediction involved a multi-class problem, we used a negative log likelihood for multi-classification as the loss function. This was set to '*mlogloss*' during the model training. To get the best split of the leaf nodes, a greedy algorithm that uses a gain function is applied to calculate the change in the objective function after adding a split. The learning process stops once an optimal depth value of a tree that maximizes the gain is reached [6].

S1 References

1. Dreiseitl S, Ohno-Machado L. Logistic regression and artificial neural network classification models: a methodology review. J Biomed Inform. 2002;35(5–6):352–9.
2. Cortes C, Vapnik V. Support-vector networks. Mach Learn. 1995;20:273–97.
3. Jain AK, Mao J, Mohiuddin KM. Artificial neural networks: A tutorial. Computer. 1996;29(3):31–44.
4. Huang S, Shen Q, Duong TQ. Artificial neural network prediction of ischemic tissue fate in acute stroke imaging. J Cereb Blood Flow Metab. 2010;30(9):1661–70.
5. Glorot X, Bordes A, Bengio Y. Deep sparse rectifier neural networks. In: Proceedings of the fourteenth international conference on artificial intelligence and statistics. JMLR Workshop and Conference Proceedings; 2011. p. 315–23.
6. Chen T, Guestrin C. Xgboost: A scalable tree boosting system. In: Proceedings of the 22nd acm sigkdd international conference on knowledge discovery and data mining. 2016. p. 785–94.
7. Friedman JH. Greedy function approximation: a gradient boosting machine. Ann Stat. 2001;1189–232.
