# Supplementary material for: Developing machine learning models to predict multi-class functional outcomes and death three months after stroke in Sweden
Source: PLoS One. 2024 May 13;19(5):e0303287. doi: 10.1371/journal.pone.0303287 (PMC11090298; doi:10.1371/journal.pone.0303287)
Supplement: S2 Method — (PDF) [file pone.0303287.s002.pdf]

S2 Method. Model Performance Metrics

Confusion Matrix: This is a table which summaries the performance of a classification model. It displays the number of true negatives (TN), false negatives (FN), true positives (TP), and false positives (FP) for each class. All the metrics that follow are obtained from the confusion matrix.

Precision (Positive Predictive Value): This is the proportion of positive class predictions that actually belong to the positive class [1]. It is given by:

$$Precision = \frac{TP}{TP + FP}$$

Recall (Sensitivity): This measures the proportion of positive predictions that were correctly predicted by a classifier [1]. It is computed as:

$$Recall = \frac{TP}{TP + FN}$$

We calculated precision and sensitivity for each class, and both their macro and weighted (adjusted for size of each outcome class) values.

Accuracy: This is the most popular performance metric for prediction models and can be calculated directly from the confusion matrix. It is the proportion of correct predictions achieved by a classifier. The formula that follows applies to both binary and multi-classification tasks.

$$Accuracy = \frac{TP + TN}{TP + FP + FN + TN},$$

where, TP, TN, FP and FN represent the number of true positive, true negative, false positive, and false negative predictions, respectively. The metric ranges from 0 (worst prediction) to 1 (best prediction).

F1-score: This is a widely applied metric in ML for both binary and multi-class classification problems. It is the harmonic mean of precision and recall [2]. Mathematically it is given as:

$$F1 = 2\frac{precision \times recall}{precision + recall} = \frac{2TP}{2TP+FP+FN}$$

The *F1*-score value ranges between 0 (both precision and recall are zero) to 1 (perfect precision and recall). To achieve accurate results for either balanced or imbalanced datasets and for multiclass tasks, the metric extends to macro, micro (same as accuracy), and weighted average *F1*-scores. An '*OvR*' approach is considered to compute the score for each class. Macro (unweighted) averaging is obtained by taking the arithmetic mean of these scores, while weighted *F1*-score is averaging adjusted for the sample size of each class in the outcome.

$$Macro\ average\ F1 = \frac{\sum_{i=1}^{k} \frac{2TP_i}{2TP_i+FP_i+FN_i}}{k},$$

$$Weighted\ average\ F1 = \frac{\sum_{i=1}^{k} \frac{2TP_i}{2TP_i+FP_i+FN_i} w_i}{W},$$

where $k = 3$, is the total number of target labels (independent, dependent, dead), w_i and W denote frequency of each class and the entire test set, respectively.

Cohen-Kappa coefficient (κ): κ was initially used to measure inter-rater level of agreement in the presence of nominal variables, but it is nowadays also applied to assess the performance of classifiers [3,4]. It shows how much a classifier performs

(the observed accuracy) compared to performance of a classifier that guesses at random (expected accuracy) according to the size of each outcome class. It ranges in the interval $[-1, +1]$, where values ≤ 0 and $+1$ represent no and perfect agreement, respectively. It is expressed as follows:

$$\kappa = \frac{P_o - P_e}{1 - P_e}$$

where, P_o is the proportion of agreement observed between true and predicted values (observed accuracy), and P_e the probability that true and false values agree by chance (expected accuracy).

***Matthews correlation coefficient (MCC)*:** MCC can be used in evaluating performance of binary classification tasks as well as multi-class cases, especially when the dataset is imbalanced [2]. It measures the strength of association between true values and predicted ones. This can be expressed as [5,6]:

$$MCC = \frac{(TP \times TN) - (FP \times FN)}{\sqrt{(TP+FP) \times (TP+FN) \times (TN+FP) \times (TN+FN)}}$$

Its value ranges from -1 (perfect disagreement between true and predicted values) to $+1$ (perfect agreement) whereas a value close to or equal 0 means no better than random predictions. MCC is symmetric, implying switching classes still leads to the same score.

***Area under the receiver operating characteristic curve (AUC-ROC)*:** AUC-ROC is a common measure of discrimination. It is obtained from the area under the ROC curve, which shows a common measure of true positives rate and true negatives rate over all possible discrimination thresholds. However, it is not recommended for imbalanced datasets [1].

$$AUC - ROC = \frac{recall + specificity}{2},$$

where,

$$Recall = \frac{TP}{TP + FN} \text{ and } specificity = \frac{TN}{TN+FP}$$

S2 References

1. Dreiseitl S, Ohno-Machado L. Logistic regression and artificial neural network classification models: a methodology review. J Biomed Inform. 2002;35(5–6):352–9.
2. Chicco D, Jurman G. The advantages of the Matthews correlation coefficient (MCC) over F1 score and accuracy in binary classification evaluation. BMC Genomics. 2020;21:1–13.
3. Cohen J. A coefficient of agreement for nominal scales. Educ Psychol Meas. 1960;20(1):37–46.
4. McHugh ML. Interrater reliability: the kappa statistic. Biochem Medica. 2012;22(3):276–82.
5. Matthews BW. Comparison of the predicted and observed secondary structure of T4 phage lysozyme. Biochim Biophys Acta BBA-Protein Struct. 1975;405(2):442–51.
6. Baldi P, Brunak S, Chauvin Y, Andersen CA, Nielsen H. Assessing the accuracy of prediction algorithms for classification: an overview. Bioinformatics. 2000;16(5):412–24.
