# Supplementary material for: Developing machine learning models to predict multi-class functional outcomes and death three months after stroke in Sweden
Source: PLoS One. 2024 May 13;19(5):e0303287. doi: 10.1371/journal.pone.0303287 (PMC11090298; doi:10.1371/journal.pone.0303287)
Supplement: S3 Method — (PDF) [file pone.0303287.s003.pdf]

S3 Method. Model Explainability

SHAPley Additive exPlanations (SHAP): SHAP is a method that was originally proposed by Lloyd Shapley to measure the contribution of each player to a game [1], and is currently helpful in ML explainability. It is a unified measure of feature importance (a combination of previous methods used for explainability) [2]. It compares the effect of including and excluding a feature in the prediction model. A SHAP value for feature i is defined as follows [2]:

$$\phi_i(P) = \sum_{S \subseteq P|\{i\}} \frac{|S|!(|P|-|S|-1)!}{|P|!} \left[f_{S \cup \{i\}}\left(x_{S \cup \{i\}}\right) - f_S\left(x_S\right) \right]$$

where, P and S respectively represent the set and subset of all features in the model, $f_{S \cup \{i\}}$ and f_S respectively denote prediction models with and without feature i, and x_S denote the input feature values in the set S.

SHAP values were presented in force plots and beeswarm plots to illustrate both local and global explainability, respectively. The former explains how each feature contributes to the prediction for a single observation (patient). Each feature forces the prediction to either increase or decrease from the SHAP baseline value to the final model prediction. The base value ($E[f(x)]$) is the average prediction of the model (log odds) based on the training data, while $f(x)$ is the final model prediction value (probability of belonging to an outcome class) of that particular observation (patient). While the latter show the importance of each feature in the prediction by combining local explanations of the model for all patients in the data set [3]. They are more informative especially for multiclass problems since they show both feature importance and direction of the relationship between the feature and the target variable. For each feature, a patient is represented by a point distributed horizontally

along the *x*-axis based on the SHAP value. Each feature in the *y*-axis is arranged in descending order of importance (higher to smaller mean absolute SHAP value). Points on the plot are coloured to differentiate high and low feature values. The horizontal axis represents the feature effect on prediction, whereby positive and negative SHAP values indicate a strong (increase) or weaker (decrease) influence of the feature on that outcome class (mRS level), respectively.

S3 References

1. Shapley LS. 17. A Value for n-Person Games [Internet]. Kuhn HW, Tucker AW, editors. Contributions to the Theory of Games (AM-28), Volume II. Princeton: Princeton University Press; 1953. p. 307–18. Available from: https://doi.org/10.1515/9781400881970-018
2. Lundberg SM, Lee SI. A unified approach to interpreting model predictions. Advances in neural information processing systems. 2017;30.
3. Lundberg SM, Erion G, Chen H, DeGrave A, Prutkin JM, Nair B, et al. From local explanations to global understanding with explainable AI for trees. Nat Mach Intell. 2020;2(1):56–67.
