# Supplementary material for: Developing machine learning models to predict multi-class functional outcomes and death three months after stroke in Sweden
Source: PLoS One. 2024 May 13;19(5):e0303287. doi: 10.1371/journal.pone.0303287 (PMC11090298; doi:10.1371/journal.pone.0303287)
Supplement: S1 Table — Summary statistics reported as number of patients (%) for binary variables, median (quartiles) for categorical variables, and mean (standard deviation) for continuous variables. (PDF) [file pone.0303287.s004.pdf]

S1 Table. Comparison of patients' characteristics at three months follow-up.

Summary statistics reported as number of patients (%) for binary variables, median (quartiles) for categorical variables, and mean (standard deviation) for continuous variables.

Variable	Patients with complete mRS at 3-months follow-up		Patients still alive at 3 months but lost to follow-up		p-value
		Missing (%)		Missing (%)	
**Number of patients (%)**	102,135 (79.6)		26,225 (20.4)		
Age (mean [SD])	75.8 [12.0]	-	72.7 [13.9]	-	<0.001
Male (%)	54,473 (53.3)	-	14,512 (55.3)		<0.001
Atrial Fibrillation (%)	30,195 (29.6)	152 (0.2)	6,895 (26.3)	152 (0.6)	<0.001
Diabetes (%)	22,355 (21.9)	214 (0.2)	6,367 (24.3)	170 (0.6)	<0.001
Previous Stroke or TIA (%)	27,787 (27.2)	186 (0.2)	7,358 (28.1)	181 (0.7)	<0.001
Smoking (%)	11,394 (11.2)	14,252 (14.0)	4,326 (16.5)	3,669 (14.0)	<0.001
Blood pressure (BP) lowering medication (%)	65,004 (63.6)	395 (0.4)	15,820 (60.3)	242 (0.9)	<0.001
Lipid-lowering drugs (%)	32,616 (31.9)	441 (0.4)	8,130 (31.0)	262 (1.0)	<0.001
Prior anticoagulation (%)	15,824 (15.5)	318 (0.3)	3,640 (13.9)	214 (0.8)	<0.001
Pre-stroke mRS (%)		5,199 (5.1)		1,240 (4.9)	<0.001
0–2 (Reference)	69,795 (68.3)		17,798 (70.6)		
3	14,772 (14.4)		4,170 (16.5)		
4	9,352 (9.2)		2,411 (9.6)		
5	3,017 (3.0)		606 (2.4)		

Variable	Patients with complete mRS at 3-months follow-up		Patients still alive at 3 months but lost to follow-up		p-value
Inpatient at time of stroke (%)	6,184 (6.1)	2 (0.0)	1,691 (6.4)	30 (0.1)	<0.001
NIHSS at arrival (median [Q1-Q3])	3.0 [1.0–8.0]	43,474 (42.6%)	3.0 [1.0–7.0]	11,552 (44.0)	<0.001
Type of stroke (%)					
Ischemic (Reference)	87,594 (85.8)	-	22,754 (86.8)	-	<0.001
Hemorrhagic	13,511 (13.2)	-	3,246 (12.4)	-	0.0003
Unspecified	1,030 (1.0)	-	225 (0.9)	-	0.0297
Wake-up stroke (%)	18,635 (18.2)	18,729 (18.3)	4,519 (17.2)	5,443 (20.8)	<0.001
Stroke alert activation (%)	35,277 (34.5)	1,319 (1.3)	8,496 (32.4)	559 (2.1)	<0.001
Ambulance service to the hospital (%)	70,786 (69.3)	8,469 (8.3)	17,674 (67.4)	2,522 (9.6)	<0.001
Hour of Admission (%)		4,021 (3.9)		1,400 (5.3)	
00–04 (Reference)	4,332 (4.2)		1,103 (4.2)		<0.001
04–08	5,852 (5.7)		1,273 (4.9)		<0.001
08–12	29,094 (28.5)		6,889 (26.3)		<0.001
12–16	27,870 (27.3)		7,184 (27.4)		<0.001
16–20	19,396 (19.0)		5,315 (20.3)		<0.001
20–24	11,570 (11.3)		3,061 (11.7)		<0.001
Day of week of admission (%)		44 (0.04)		154 (0.6)	
Sunday (Reference)	13,214 (12.9)		3,352 (12.8)		<0.001
Monday	16,255 (15.9)		4,143 (15.8)		<0.001
Tuesday	15,377 (15.1)		4,022 (15.3)		<0.001
Wednesday	15,147 (14.8)		3,818 (14.6)		<0.001
Thursday	14,740 (14.4)		3,855 (14.7)		<0.001
Friday	14,446 (14.1)		3,685 (14.1)		<0.001

Variable	Patients with complete mRS at 3-months follow-up		Patients still alive at 3 months but lost to follow-up		p-value
Saturday	12,912 (12.6)		3,196 (12.2)		<0.001
