# Supplementary material for: Developing machine learning models to predict multi-class functional outcomes and death three months after stroke in Sweden
Source: PLoS One. 2024 May 13;19(5):e0303287. doi: 10.1371/journal.pone.0303287 (PMC11090298; doi:10.1371/journal.pone.0303287)
Supplement: S2 Table — The best hyperparameter values obtained from the algorithm are presented in the last column of the table. Meaning of these hyperparameters can be obtained from the SKlearn library ([1] reference for S2 Table). (PDF) [file pone.0303287.s005.pdf]

S2 Table. Model hyperparameters search space (selected based on best guess). The best hyperparameter values obtained from the algorithm are presented in the last column of the table. Meaning of these hyperparameters can be obtained from the SKlearn library [1].

Model (Multi-class)	Hyperparameter	Search space	Best parameter
LR*	multi_class	-	‘multinomial’
	penalty	-	‘none’
	class_weight	-	‘balanced’
	solver	-	‘saga’
	max_iter	-	10000
	n_jobs	-	-1
	random_state	-	48572
	dual	-	*False*
	tol	-	*0.0001*
	C	-	*1.0*
	fit_intercept	-	*True*
	intercept_scaling	-	*1*
	verbose	-	*0*
	warm_start	-	*False*
	l1_ratio	-	*None*
XGBoost	max_depth	list(range(5, 9))	6
	learning_rate**	0.2	0.2
	n_estimators	[120, 80, 150, 100]	150
	objective**	‘multi:softprob’	‘multi:softprob’
	gamma	[0.001, 1, 0.1]	0.001
	min_child_weight	[10, 20, 15]	10
	subsample	[0.5, 0.7]	0.7
	colsample_bytree**	0.5	0.5

Model (Multi-class)	Hyperparameter	Search space	Best parameter
	colsample_bylevel**	0.5	0.5
	colsample_bynode**	0.5	0.5
	reg_alpha	[0.001, 0.01, 1]	1
	reg_lambda	[2, 1, 1.5, 5]	1
	seed**	48572	48572
	scale_pos_weight***	-	-
	eval_metric**	‘mlogloss’	‘mlogloss’
	early_stopping_rounds **		10
	base_score		*None*
	booster		*‘gbtree’*
	enable_categorical		*False*
	importance_type		*None*
	tree_method		*None*
	use_label_encoder		*False*
	max_leaves		*None*
	grow_policy		*None*
	max_bin		*None*
	verbosity		*3*
	monotone_constraints		*None*
	nthread		*None*
SVM	C	[25, 50, 20, 10]	50
	kernel	[‘sigmoid’, ‘rbf’]	‘rbf’
	gamma	[‘scale’, 1, 0.01, 0.1, 0.001]	0.01
	class_weight**	‘balanced’	‘balanced’
	probability**	True	True
	random_state**	48572	48572
	coef0		*0.0*

Model (Multi-class)	Hyperparameter	Search space	Best parameter
	shrinking		*True*
	tol		*0.001*
	cache_size		*200*
	verbose		*False*
	max_iter		*-1*
	decision_function_shape		*'ovr'*
	break_ties		*False*
ANN	hidden_layer_sizes	[(50,50), (200, 200), (10,10), (100,100)]	(10,10)
	alpha	[0.0001, 0.00001, 1, 0.01]	0.0001
	batch_size	[60, 1500, 500, 1000]	60
	random_state**	48572	48572
	max_iter	[10000, 20000, 15000]	10000
	n_iter_no_change	[10, 300, 100, 30]	100
	early_stopping**	True	True
	validation_fraction**	0.2	0.2
	learning_rate	['constant', 'adaptive']	'constant'
	activation		*'relu'*
	solver		*'adam'*
	learning_rate_init		*0.001*
	power_t		*0.5*
	shuffle		*True*
	tol		*0.0001*
	verbose		*False*
	warm_start		*False*
	momentum		*0.9*
	nesterovs_momentum		*True*

Model (Multi-class)	Hyperparameter	Search space	Best parameter
	beta_1		*0.9*
	beta_2		*0.999*
	epsilon		*0.00000007*
	max_fun		*15000*

*We explicitly set the parameters to train a main effect multinomial logistic regression (mLR) model. No tuning was performed for this model. **Values of these hyperparameters were fixed during the training. ***Hyperparameter for binary problems to account for an imbalance dataset, thus instead, we used the 'compute_sample_weight' function from SKlearn library [1]. *Italic* indicates hyperparameters set to default.

S2 Table Reference

1. Pedregosa F, Varoquaux G, Gramfort A, Michel V, Thirion B, Grisel O, et al. Scikit-learn: Machine Learning in Python. J Mach Learn Res. 2011;12(85):2825–30.
