# Supplementary material for: Developing machine learning models to predict multi-class functional outcomes and death three months after stroke in Sweden
Source: PLoS One. 2024 May 13;19(5):e0303287. doi: 10.1371/journal.pone.0303287 (PMC11090298; doi:10.1371/journal.pone.0303287)
Supplement: S3 Table — A. Estimates (with 95% confidence interval (CI)) for the Model Performance Metrics in the test set. B. Estimates (with 95% confidence interval (CI)) for the Model Performance Metrics in the training set). (PDF) [file pone.0303287.s006.pdf]

S3A Table. Estimates (with 95% confidence interval (CI)) for the Model Performance Metrics in the test set.

Performance indicators [95% CI]/ Models	Multinomial LR	Multinomial LR with interaction terms	XGBoost	SVM	ANN
Accuracy	0.689[4] [0.684, 0.695]	0.690[3] [0.684, 0.696]	0.694[2] [0.688, 0.699]	0.688[5] [0.683, 0.694]	0.698[1] [0.693, 0.704]
Cohen-Kappa coefficient	0.518[3] [0.509, 0.526]	0.517[4] [0.508, 0.526]	0.526[1] [0.517, 0.534]	0.516[5] [0.507, 0.524]	0.526[1] [0.517, 0.535]
Matthews correlation coefficient	0.522[4] [0.514, 0.531]	0.523[3] [0.513, 0.531]	0.527[1] [0.519, 0.536]	0.522[4] [0.514, 0.531]	0.527[1] [0.518, 0.536]
F1-score					
mRS 0–2	0.796[2] [0.790, 0.801]	0.795[3] [0.789, 0.800]	0.794[4] [0.788, 0.800]	0.797[1] [0.791, 0.803]	0.793[5] [0.788, 0.799]
mRS 3–5	0.554[3] [0.545, 0.563]	0.552[4] [0.542, 0.562]	0.577[2] [0.568 , 0.586]	0.541[5] [0.532, 0.551]	0.603[1] [0.594, 0.611]
mRS 6	0.667[3] [0.656, 0.676]	0.669[2] [0.658, 0.679]	0.671[1] [0.660, 0.680]	0.667[3] [0.657, 0.677]	0.657[5] [0.646, 0.668]
Macro average	0.672[3] [0.666, 0.678]	0.672[3] [0.666, 0.678]	0.681[2] [0.675, 0.687]	0.668[5] [0.663, 0.674]	0.685[1] [0.678, 0.691]
Weighted average	0.681[3] [0.675, 0.687]	0.680[4] [0.674, 0.686]	0.690[2] [0.684, 0.695]	0.677[5] [0.671, 0.683]	0.696[1] [0.690, 0.702]
Sensitivity (Recall)					
mRS 0–2	0.848[3] [0.842, 0.855]	0.859[2] [0.853, 0.865]	0.826[5] [0.818, 0.833]	0.865[1] [0.859, 0.872]	0.830[4] [0.823, 0.837]
mRS 3–5	0.489[3] [0.479, 0.500]	0.483[4] [0.474, 0.494]	0.534[2] [0.524, 0.545]	0.464[5] [0.455, 0.475]	0.598[1] [0.588, 0.609]
mRS 6	0.710[2] [0.697, 0.722]	0.701[4] [0.688, 0.713]	0.702[3] [0.689, 0.715]	0.713[1] [0.700, 0.725]	0.607[5] [0.592, 0.620]
Macro average	0.682[2] [0.677, 0.688]	0.681[3] [0.675, 0.687]	0.687[1] [0.681, 0.693]	0.681[3] [0.675, 0.687]	0.678[5] [0.672, 0.684]
Weighted average	0.689[4] [0.684, 0.695]	0.690[3] [0.684, 0.696]	0.694[2] [0.688, 0.699]	0.688[5] [0.683, 0.694]	0.698[1] [0.693, 0.704]
Precision (PPV)					
mRS 0–2	0.749[3] [0.742, 0.757]	0.739[4] [0.732, 0.747]	0.765[1] [0.758, 0.773]	0.739[4] [0.732, 0.747]	0.760[2] [0.753, 0.768]

mRS 3–5	0.639[3] [0.627, 0.650]	0.644[2] [0.631, 0.655]	0.628[4] [0.618, 0.639]	0.650[1] [0.638, 0.661]	0.608[5] [0.597, 0.618]
mRS 6	0.629[4] [0.616, 0.641]	0.639[3] [0.626, 0.651]	0.641[2] [0.629, 0.654]	0.626[5] [0.613, 0.638]	0.717[1] [0.705, 0.730
Macro average	0.672[4] [0.666, 0.678]	0.674[3] [0.667, 0.680]	0.678[2] [0.672, 0.684]	0.672[4] [0.666, 0.678]	0.695[1] [0.689, 0.701]
Weighted average	0.683[3] [0.678, 0.689]	0.683[3] [0.677, 0.689]	0.689[2] [0.683, 0.695]	0.682[5] [0.677, 0.689]	0.696[1] [0.690, 0.702]

[1,2,3,4,5] are the ranking of the model prediction performances based on the respective metrics. PPV; positive predictive value.

S3B Table. Estimates (with 95% confidence interval (CI)) for the Model Performance Metrics in the training set.

Performance indicators [95% CI]/ Models	Multinomial LR	Multinomial LR with interaction terms	XGBoost	SVM	ANN
Accuracy	0.687^{5} [0.683, 0.690]	0.690^{4} [0.686, 0.693]	0.704^{1} [0.701, 0.707]	0.693^{3} [0.690, 0.696]	0.702^{2} [0.699, 0.705]
Cohen-Kappa coefficient	0.514^{5} [0.508, 0.519]	0.517^{4} [0.512, 0.522]	0.541^{1} [0.536, 0.546]	0.522^{3} [0.516, 0.526]	0.530^{2} [0.525, 0.535]
Matthews correlation coefficient	0.517^{5} [0.512, 0.523]	0.522^{4} [0.517, 0.527]	0.543^{1} [0.538, 0.548]	0.529^{3} [0.524, 0.534]	0.533^{2} [0.528, 0.537]
F1-score					
mRS 0–2	0.792^{5} [0.788, 0.795]	0.793^{4} [0.790, 0.797]	0.796^{2} [0.793, 0.800]	0.796^{2} [0.792, 0.799]	0.799^{1} [0.795, 0.802]
mRS 3–5	0.553^{4} [0.548, 0.559]	0.554^{3} [0.549, 0.560]	0.592^{2} [0.587, 0.597]	0.551^{5} [0.546, 0.556]	0.605^{1} [0.600, 0.610]
mRS 6	0.665^{4} [0.660, 0.671]	0.670^{3} [0.664, 0.675]	0.689^{1} [0.684, 0.695]	0.676^{2} [0.670, 0.681]	0.650^{5} [0.644, 0.656]
Macro average	0.670^{5} [0.667, 0.674]	0.672^{4} [0.669, 0.676]	0.693^{1} [0.689, 0.696]	0.674^{3} [0.671, 0.678]	0.685^{2} [0.681, 0.688]
Weighted average	0.679^{5} [0.675, 0.682]	0.681^{4} [0.677, 0.684]	0.700^{1} [0.696, 0.703]	0.682^{3} [0.678, 0.685]	0.697^{2} [0.694, 0.700]
Sensitivity (Recall)					
mRS 0–2	0.841^{4} [0.837, 0.845]	0.855^{3} [0.851, 0.859]	0.825^{5} [0.821, 0.828]	0.874^{1} [0.870, 0.877]	0.861^{2} [0.857, 0.864]
mRS 3–5	0.492^{3} [0.486, 0.498]	0.488^{4} [0.482, 0.494]	0.549^{2} [0.543, 0.555]	0.474^{5} [0.468, 0.480]	0.590^{1} [0.584, 0.596]
mRS 6	0.707^{2} [0.699, 0.714]	0.700^{4} [0.693, 0.707]	0.724^{1} [0.718, 0.731]	0.703^{3} [0.696, 0.711]	0.579^{5} [0.571, 0.587]
Macro average	0.680^{4} [0.676, 0.683]	0.681^{3} [0.678, 0.684]	0.699^{1} [0.696, 0.703]	0.684^{2} [0.680, 0.687]	0.676^{5} [0.673, 0.680]
Weighted average	0.687^{5} [0.683, 0.690]	0.690^{4} [0.686, 0.693]	0.704^{1} [0.701, 0.707]	0.693^{3} [0.690, 0.696]	0.702^{2} [0.699, 0.705]
Precision (PPV)					
mRS 0–2	0.748^{2} [0.744, 0.753]	0.740^{4} [0.735, 0.745]	0.770^{1} [0.766, 0.775]	0.730^{5} [0.726, 0.735]	0.745^{3} [0.741, 0.750]

mRS 3–5	0.632[4] [0.626, 0.639]	0.641[3] [0.634, 0.647]	0.642[2] [0.636, 0.648]	0.659[1] [0.652, 0.666]	0.621[5] [0.616, 0.627]
mRS 6	0.628[5] [0.621, 0.635]	0.642[4] [0.635, 0.649]	0.657[2] [0.650, 0.664]	0.650[3] [0.643, 0.657]	0.741[1] [0.734, 0.748]
Macro average	0.669[5] [0.666, 0.673]	0.674[4] [0.671, 0.678]	0.690[2] [0.687, 0.693]	0.680[3] [0.676, 0.683]	0.703[1] [0.699, 0.706]
Weighted average	0.681[5] [0.677, 0.684]	0.683[4] [0.680, 0.686]	0.700[1] [0.696, 0.703]	0.687[3] [0.684, 0.691]	0.700[1] [0.696, 0.703]

[1,2,3,4,5] are the ranking of the model prediction performances based on the respective metrics. PPV; positive predictive value.
