# Supplementary material for: Developing machine learning models to predict multi-class functional outcomes and death three months after stroke in Sweden
Source: PLoS One. 2024 May 13;19(5):e0303287. doi: 10.1371/journal.pone.0303287 (PMC11090298; doi:10.1371/journal.pone.0303287)
Supplement: S4 Table — The estimates indicate how a unit increase in the covariate is associated with the odds of belonging to a particular mRS level, keeping other covariates fixed. OR = 1 means that the covariate is not associated with odds of the corresponding mRS level, OR > 1 means that there is increased occurrence of the corresponding mRS level as the covariate increases (or at that level of covariate), and OR < 1 indicates that the corresponding mRS level is less likely to occur as the covariate increases (or at that level of covariate). (PDF) [file pone.0303287.s007.pdf]

S4 Table. Estimated odds ratios (exp(regression slope coefficients)) from the main-effects multinomial LR model. The estimates indicate how a unit increase in the covariate is associated with the odds of belonging to a particular mRS level, keeping other covariates fixed. $OR = 1$ means that the covariate is not associated with odds of the corresponding mRS level, $OR > 1$ means that there is increased occurrence of the corresponding mRS level as the covariate increases (or at that level of covariate), and $OR < 1$ indicates that the corresponding mRS level is less likely to occur as the covariate increases (or at that level of covariate).

Covariates / Coefficients	mRS level		
	0: Independent	1: Dependent	2: Dead
Age	0.958	1.004	1.040
Sex	1.125	0.820	1.085
Inpatient at time of Stroke			
Yes	0.454	0.885	2.491
Unknown	1.000	0.936	1.069
Hour of Admission			
040000to075959			
Yes	0.984	0.982	1.035
Unknown	0.981	1.028	0.991
080000to115959			
Yes	1.060	1.016	0.928
Unknown	0.981	1.028	0.991
120000to155959			
Yes	1.001	1.099	0.909
Unknown	0.981	1.028	0.991
160000to195959			
Yes	0.963	1.099	0.945
Unknown	0.981	1.028	0.991
200000to235959			
Yes	0.939	1.075	0.992
Unknown	0.981	1.028	0.991
Day of week of Admission			
Monday			
Yes	1.059	0.983	0.960
Unknown	1.366	1.246	0.588
Tuesday			
Yes	1.055	0.964	0.983
Unknown	1.366	1.246	0.588
Wednesday			
Yes	1.054	0.987	0.961

Covariates / Coefficients	mRS level		
	0: Independent	1: Dependent	2: Dead
Unknown	1.366	1.246	0.588
Thursday			
Yes	1.069	0.959	0.976
Unknown	1.366	1.246	0.588
Friday			
Yes	1.045	0.950	1.007
Unknown	1.366	1.246	0.588
Saturday			
Yes	1.020	0.961	1.020
Unknown	1.366	1.246	0.588
Hypertension			
Yes	1.000	1.023	0.977
Unknown	0.888	0.999	1.127
Atrial Fibrillation (AF)			
Yes	0.890	0.990	1.135
Unknown	0.670	0.850	1.757
Diabetes			
Yes	0.712	1.156	1.214
Unknown	1.222	0.780	1.049
Previous Stroke-TIA			
Yes	0.807	1.149	1.078
Unknown	1.020	1.043	0.940
Prior-anticoagulation			
Yes	0.912	1.003	1.094
Unknown	0.578	1.135	1.524
Prior mRS			
3	0.247	2.006	2.019
4	0.088	2.487	4.579
5	0.021	3.960	12.167
Unknown	0.207	1.238	3.897
NIHSS at admission	0.882	1.005	1.128
Type of Stroke:			
Haemorrhage	0.541	1.006	1.838
Unknown	0.882	0.805	1.407
Smoking			
Yes	0.768	1.098	1.186
Unknown	0.799	0.938	1.334
Lipid			
Yes	1.147	0.984	0.886
Unknown	1.037	0.609	1.585

Covariates / Coefficients	mRS level		
	0: Independent	1: Dependent	2: Dead
Wake-up Stroke<br>Yes<br>Unknown	 1.021<br>0.629	 1.041<br>1.148	 0.941<br>1.386
Stroke Alert<br>Yes<br>Unknown	 1.338<br>1.143	 0.972<br>1.018	 0.769<br>0.859
Ambulance<br>Yes<br>Unknown	 0.574<br>0.829	 1.154<br>1.129	 1.508<br>1.068
