# Supplementary figures and images for: Developing machine learning models to predict multi-class functional outcomes and death three months after stroke in Sweden
Source: PLoS One. 2024 May 13;19(5):e0303287. doi: 10.1371/journal.pone.0303287 (PMC11090298; doi:10.1371/journal.pone.0303287)

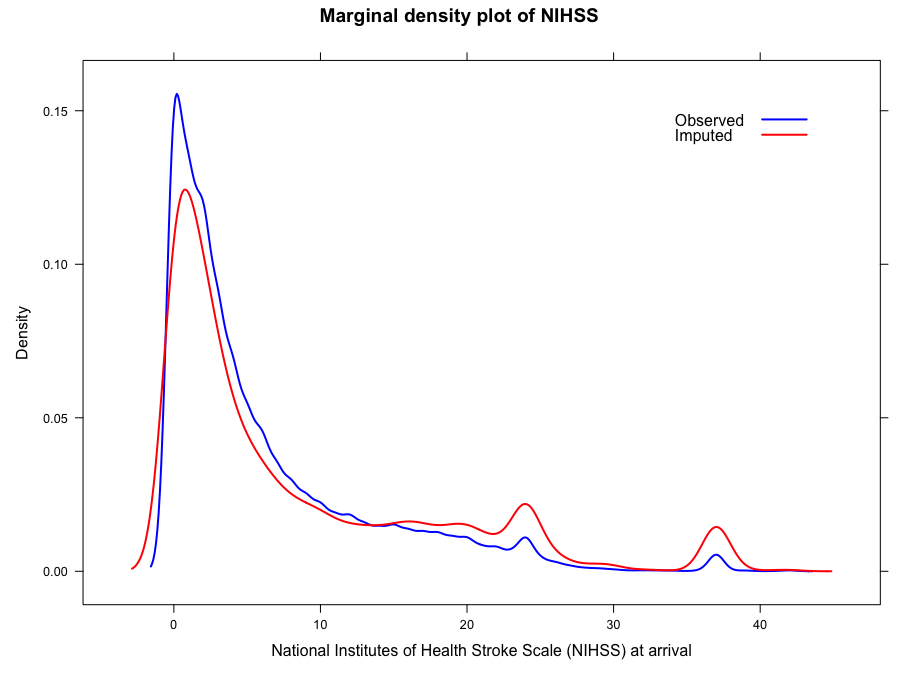

Supplement: S1 Fig — (TIFF) [file pone.0303287.s008.tiff]

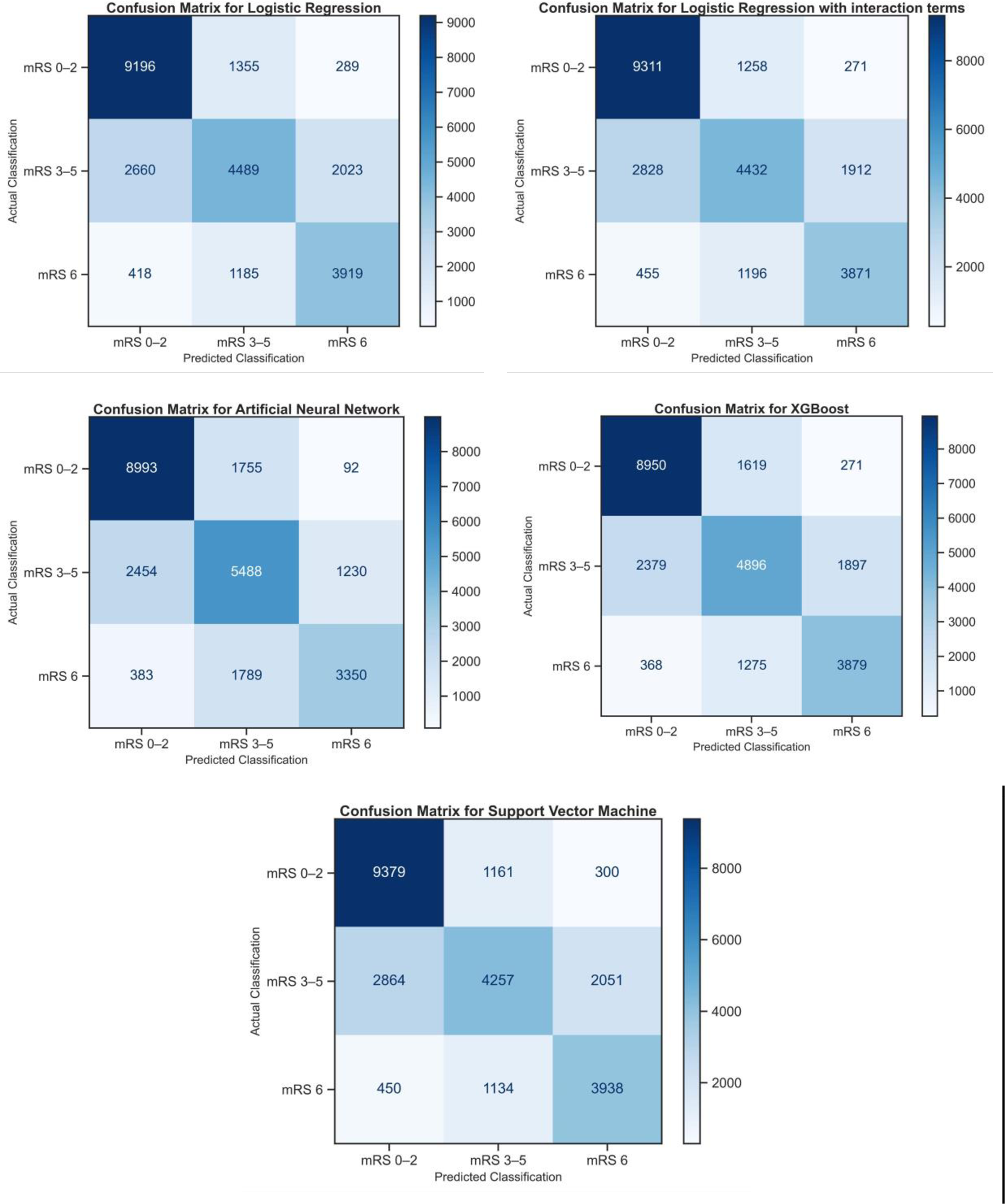

Supplement: S2 Fig — (TIFF) [file pone.0303287.s009.tiff]

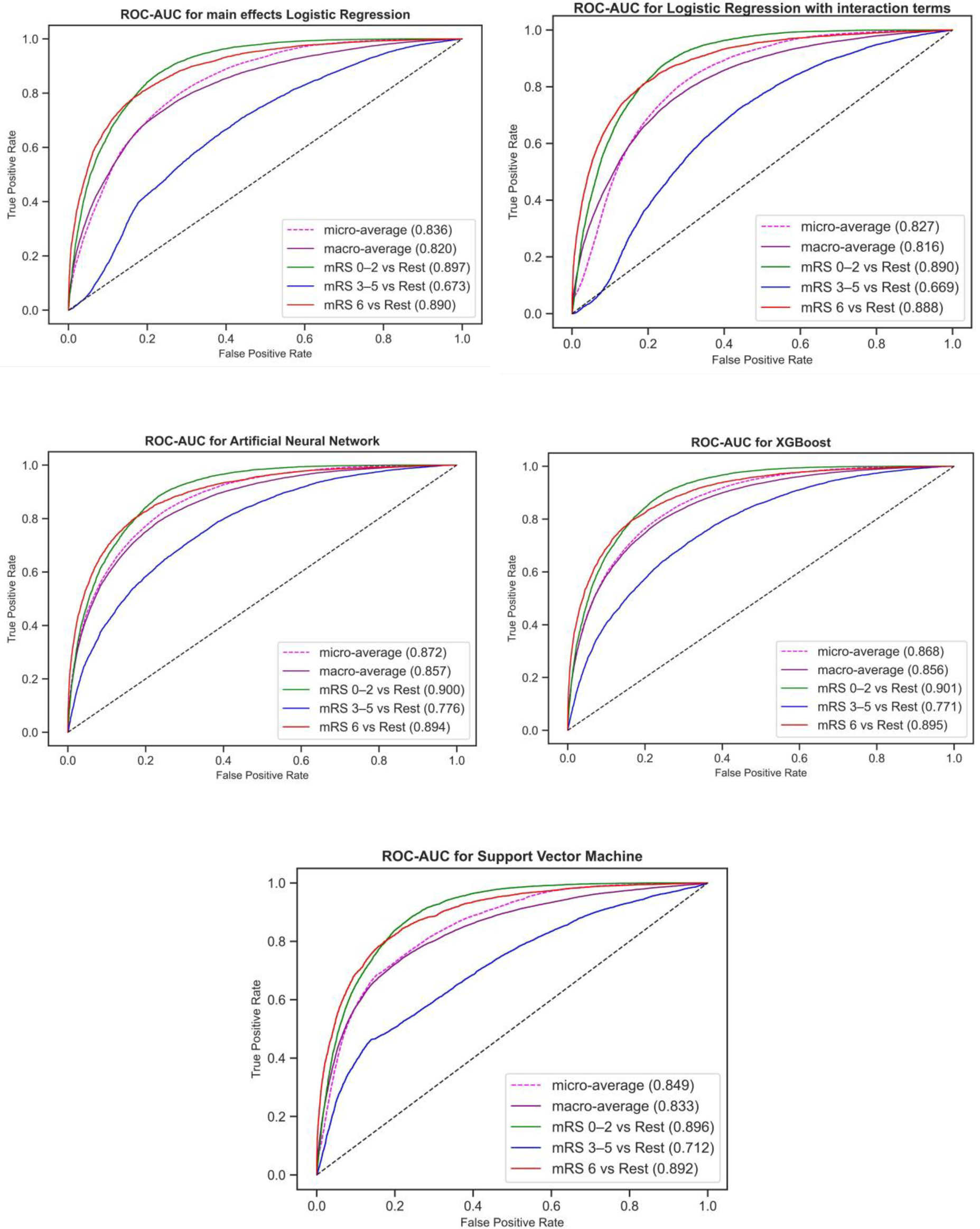

Supplement: S3 Fig — Macro-average is the arithmetic mean of AUC of each target class (equal contribution of weight for each class) while Micro-average is averaging that accounts for the size of each target class. Since micro-average AUC-ROC is dominated by the highest frequency class, macro-averaging becomes an alternative when the performance on all the classes is equally important. (TIFF) [file pone.0303287.s010.tiff]

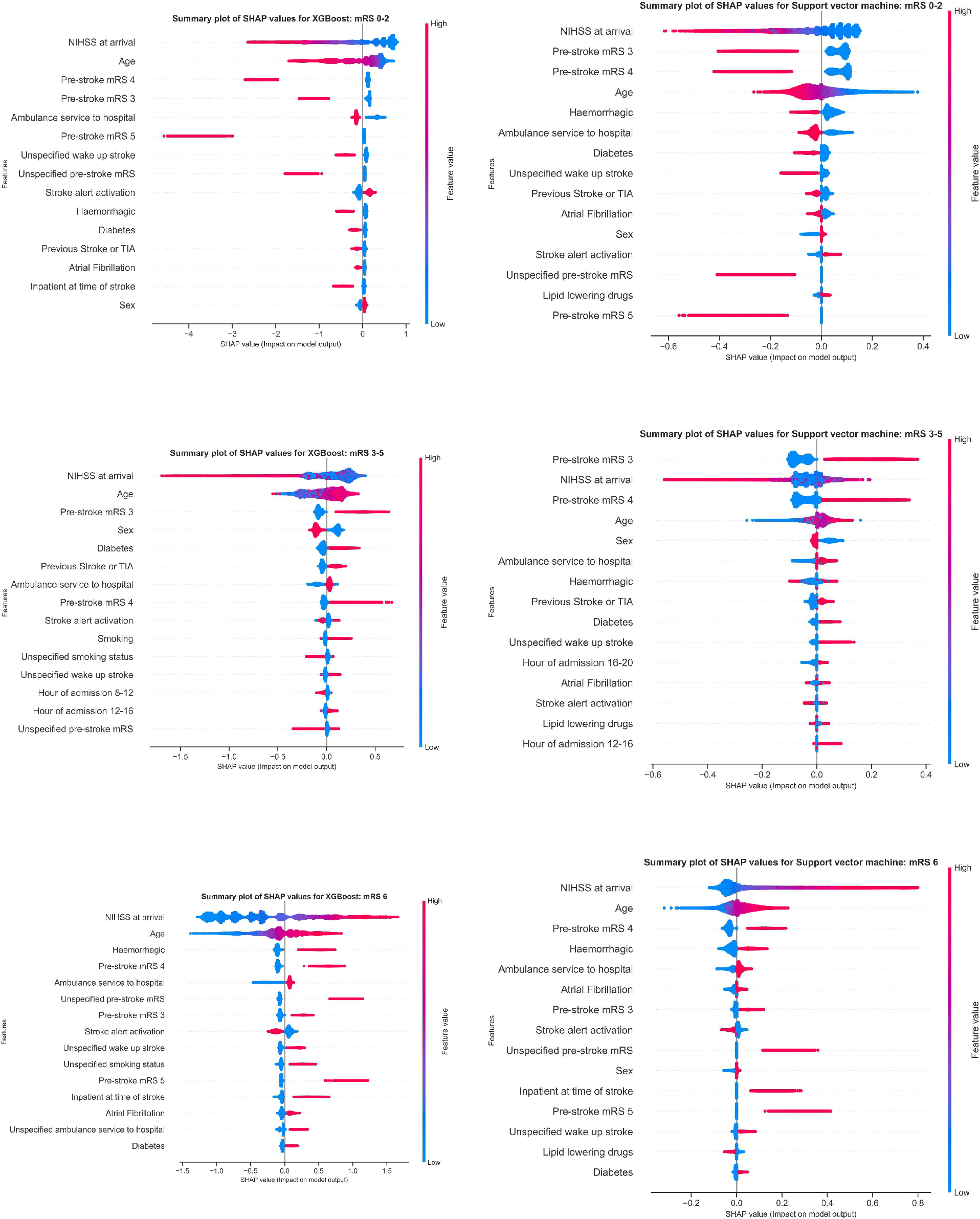

Supplement: S4 Fig — (TIFF) [file pone.0303287.s011.tiff]
